# Primary Sensory and Motor Cortex Excitability Are Co-Modulated in Response to Peripheral Electrical Nerve Stimulation

**DOI:** 10.1371/journal.pone.0051298

**Published:** 2012-12-05

**Authors:** Siobhan M. Schabrun, Michael C. Ridding, Mary P. Galea, Paul W. Hodges, Lucinda S. Chipchase

**Affiliations:** 1 The University of Queensland, NHMRC Centre of Clinical Research Excellence in Spinal Pain, Injury and Health and School of Health and Rehabilitations Sciences, Brisbane, Queensland, Australia; 2 The Robinson Institute, School of Paediatrics and Reproductive Health, The University of Adelaide, South Australia, Australia; 3 Rehabilitation Sciences Research Centre, The University of Melbourne, Victoria, Australia; The University of Western Ontario, Canada

## Abstract

Peripheral electrical stimulation (PES) is a common clinical technique known to induce changes in corticomotor excitability; PES applied to induce a tetanic motor contraction increases, and PES at sub-motor threshold (sensory) intensities decreases, corticomotor excitability. Understanding of the mechanisms underlying these opposite changes in corticomotor excitability remains elusive. Modulation of primary sensory cortex (S1) excitability could underlie altered corticomotor excitability with PES. Here we examined whether changes in primary sensory (S1) and motor (M1) cortex excitability follow the same time-course when PES is applied using identical stimulus parameters. Corticomotor excitability was measured using transcranial magnetic stimulation (TMS) and sensory cortex excitability using somatosensory evoked potentials (SEPs) before and after 30 min of PES to right abductor pollicis brevis (APB). Two PES paradigms were tested in separate sessions; PES sufficient to induce a tetanic motor contraction (30–50 Hz; strong motor intensity) and PES at sub motor-threshold intensity (100 Hz). PES applied to induce strong activation of APB increased the size of the N_20_-P_25_ component, thought to reflect sensory processing at cortical level, and increased corticomotor excitability. PES at sensory intensity decreased the size of the P25-N33 component and reduced corticomotor excitability. A positive correlation was observed between the changes in amplitude of the cortical SEP components and corticomotor excitability following sensory and motor PES. Sensory PES also increased the sub-cortical P_14_-N_20_ SEP component. These findings provide evidence that PES results in co-modulation of S1 and M1 excitability, possibly due to cortico-cortical projections between S1 and M1. This mechanism may underpin changes in corticomotor excitability in response to afferent input generated by PES.

## Introduction

Peripheral electrical stimulation (PES) is used in clinical settings for a diverse range of applications from facilitation of voluntary muscle contraction to management of pain in neurological and musculoskeletal conditions. Although evidence for clinical effectiveness is growing, the physiological bases for such effects are not completely understood. In terms of PES interventions that change muscle activation, most investigations have focussed on changes at the muscle or spinal motoneurones. For instance, PES-induced muscle contractions enhance oxidative capacity, increase number of capillaries and transform muscle fibre type within a muscle [1], [2]. Yet, PES can also induce plastic change in motor regions of the human cortex (for review see [3]). Corticomotor excitability, assessed by transcranial magnetic stimulation (TMS), is increased following PES at intensities sufficient to produce muscle contraction, but decreased when PES is applied at lower intensities that are sufficient to evoke sensation without muscle contraction [4]. The mechanisms responsible for these intensity-dependent differences in the direction of the changes in excitability are not known.

Afferent input is a powerful driver of plastic change in M1. Functional and anatomical interactions exist between primary sensory (S1) and primary motor (M1) cortical areas. For example, long term potentiation (LTP) is evident in neurons of the motor cortex following tetanic stimulation of S1 [5], and ablation of S1 impairs learning, but not retention, of new motor skills [6]. These findings suggest an important role of input from S1 to M1 in modulation of M1 excitability and motor learning. Such a mechanism may underlie altered M1 excitability with PES. Specifically, excitability changes in M1 with PES may be secondary to activation of, or changes in, S1.

Previous studies have examined the effect of PES using a range of stimulus parameters on excitability of *either* M1 or S1. In relation to S1, the amplitude of short-latency components of the somatosensory evoked potential (SEP), thought to be related to cortical processing in S1 (e.g. N_20_-P_25_-N_33_), is decreased in response to high frequency PES (100–200 Hz) at intensities ranging from below motor threshold to that sufficient to induce a muscle twitch [7]–[9]. The amplitude of motor evoked potentials (MEPs) from TMS applied to M1 are decreased following PES at similar frequencies (100 Hz), but with weaker stimulation intensity [4]. No study has investigated the effect of PES applied at an intensity and frequency sufficient to induce a tetanic motor response (strong motor intensity; 30–50 Hz) on responses related to function of the primary sensory cortex (S1), despite use of this paradigm in clinical settings. The heterogeneous approach to experimental study of stimulus parameters, and failure to examine both S1 and M1 concurrently, mean it is not yet possible to conclude whether changes at S1 present a possible candidate mechanism underpinning changes in motor output following PES.

Here we compared the response of S1 and M1 to PES paradigms applied either at an intensity sufficient to evoke a contraction of the stimulated muscle or at an intensity sufficient to induce sensory stimulation, but below motor threshold.

## Materials and Methods

### Ethics Statement

All procedures were approved by the Human Research Ethics Committee at The University of Queensland and conformed to the Declaration of Helsinki.

### Participants

Thirteen healthy individuals (nine female, four male; age 27±9 years; mean ± standard deviation) gave informed and written consent to participate in the study. Participants had no history of neurological or upper limb conditions and completed a TMS safety screen prior to commencement [10].

### Electromyography (EMG)

EMG activity was recorded using disposable silver/silver chloride surface electrodes from the right abductor pollicis brevis muscle (APB). The reference electrode was placed over the metacarpophalangeal joint and the active electrode over the muscle motor point. EMG signals were amplified 1000 x, filtered between 20–1000 Hz and sampled at 2000 Hz using Signal3 software and a Micro1401 data acquisition system (Cambridge Electronic Design, Cambridge, UK).

### TMS of the Primary Motor Cortex

TMS was applied using a Magstim 200 stimulator (Magstim Co. Ltd, Dyfed, UK) with a figure-of-eight shaped coil (external wing diameter, 7 cm). The coil was held over the left hemisphere at an angle of 45° to the sagittal with the handle posterior. This coil orientation is optimal for stimulation of the hand region of the motor cortex. The optimal scalp site to evoke motor evoked potentials (MEPs) in right APB was established and marked on the scalp. Resting motor threshold (rMT) was identified as the minimum stimulator intensity at which 5 out of 10 stimuli applied at the optimal scalp site evoked a response with a peak-to-peak amplitude of at least 50 µV in the target muscle. MEPs were recorded from right ABP with stimulator output at 120% rMT. All TMS procedures adhered to the TMS checklist for methodological quality [11].

### Brachial Plexus Stimulation

Electrical stimuli of 200 µs duration were applied with a constant current stimulator (DS7A, Digitimer Ltd, Welwyn Garden City, UK) applied to the brachial plexus to evaluate changes in excitability at the muscle and neuromuscular junction. The active electrode was positioned in the supraclavicular fossa (Erb’s point) and the reference electrode over the acromion. Stimulus intensity was set 50% above the intensity required to elicit a maximal compound muscle action potential (M_max_) in the APB muscle at rest.

### Electroencephalography (EEG) Recordings - SEP

SEPs were obtained by stimulation of the median nerve at the wrist. EEG was recorded over the approximate location of the hand area of the primary sensory cortex using gold plated cup electrodes (C3’ [2 cm posterior to C3] and referenced to Fz) [12]. Electrode impedance was maintained below 5 kΩ. Additional recording electrodes were placed over the cervical spine (C7) and Erb’s point (supraclavicular fossa and acromion) in order to track the afferent volley in the spine and periphery. EEG signals were amplified 50000x, filtered 5–500 Hz and sampled at 1000 Hz using the Micro1401 data acquisition system.

A constant current stimulator was used to deliver electrical stimuli of 1-ms duration to the median nerve at a rate of 2 Hz (maximum current of 1 A). A 20% variance was incorporated into the stimulus frequency to avoid accommodation. Stimulus intensity was set at 3× perceptual threshold. This intensity was considered comfortable by all participants and was sufficient to evoke a visible muscle twitch in APB. Where necessary, the stimulus intensity was adjusted to ensure the size of the peripheral volley (recording at Erb’s point) remained constant throughout the experiment. Two blocks of 500 stimuli were recorded and averaged off line for analysis.

### PES Interventions

Each subject participated in two sessions separated by at least 72 hours. On each occasion, a different electrical stimulation intervention was administered to the right APB. The order in which participants received the two electrical stimulation paradigms was randomised. Each intervention lasted for 30 min and was delivered using a monophasic waveform with a pulse duration of 0.1 ms (Chattanooga Intelect Advanced therapy system, OPC Health, Melbourne, Australia). Habituation to the stimulus was monitored and, where necessary, the intensity adjusted to maintain a consistent motor or sensory response. To control for attention participants were directed to focus on the stimulation and verbal reminders were given at 5 min intervals.

The two interventions were:

1. *Motor Movement:* To mimic a voluntary contraction in the APB muscle, current was delivered at 30 Hz with a ramped intensity with six periods of stimuli applied per minute (4 s on: 6 s off periods). Stimulus intensity set at that sufficient to induce a mid-range thumb abduction.
2. *Sensory 100 Hz:* Intensity of electrical stimulation was set at that where the subject first reported perception of the stimulus, and delivered at a frequency of 100 Hz. This intensity was sufficient to produce a mild cutaneous tingling over the APB muscle, but without muscle contraction.

### Experimental Protocol

Participants were positioned comfortably in an armchair with their right arm relaxed and supported on an arm rest for the duration of the experiment. Fifteen baseline MEPs, 4 M_max_ measures and 2 blocks of SEP measures (500 stimuli each) were recorded. Following this, one of the PES paradigms was applied to the right APB. After completion of the stimulation period, measures of MEPs, M_max_ and SEPs were repeated.

### Data and Statistical Analyses

MEPs and M_max_ were analysed as peak-to-peak amplitudes. Each parameter was assessed with a separate two-way repeated measures analysis of variance (ANOVA) with factors time (pre/post PES) and condition (sensory PES/motor PES). To account for any activity-dependent changes in muscle fibre action potentials resulting from the PES interventions, statistical analysis was also performed with MEP amplitudes expressed as a proportion of M_max_ amplitude.

**Figure 1:**
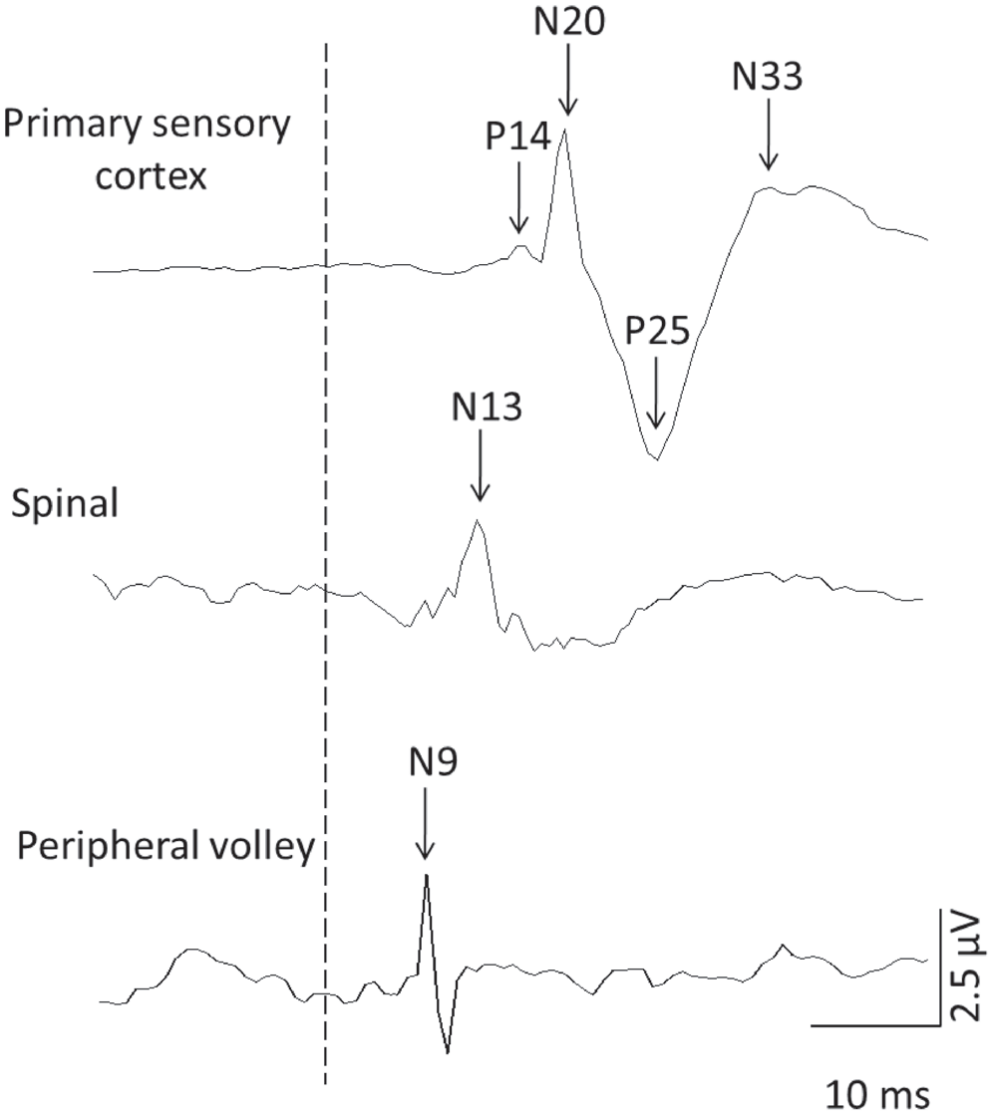
Raw data from a representative subject demonstrating the SEP components used for analysis of conduction and processing of the afferent volley at the primary sensory cortex, brainstem and the peripheral volley recorded at Erb’s point. The dotted line represents the time of stimulation.

SEP parameters were analysed as peak-to-peak amplitudes for the components: P_14_-N_20_, N_20_-P_25_, P_25_-N_33_ and the spinal (N_13_) and peripheral (N_9_) volley. Latencies were calculated as the time from stimulus onset to N_20_, N_9_ and N_13_. An example of the SEP components is presented in Figure 1. Amplitudes and latencies were analysed using separate two-way repeated measures ANOVA with factors time (pre/post PES) and condition (sensory PES/motor PES) for each parameter.

Linear regression analyses were performed to determine whether peripheral electrical stimulation induced changes in corticomotor excitability (increased/decreased MEP amplitude) were associated with changes in the amplitude of cortical (N_20_-P_25_ and P_25_-N_33_) components of the SEP. A linear regression was calculated using the pre-post change scores, calculated as 100– (MEP or SEP pre/MEP or SEP post * 100) for each measure. As findings from the repeated measures ANOVA indicated that M1 and S1 co-modulate in response to both motor and sensory PES, linear regression was calculated with data averaged over PES conditions.

Where appropriate, post-hoc tests were performed using Holm-Sidak pair-wise comparisons. Significance was set at 5%.

## Results

There was no change in M_max_ across time with either PES intervention (time p = 0.94, condition p = 0.26, Interaction time × condition p = 0.47). As M_max_ did not change, results obtained using raw MEP amplitudes and those normalised to M_max_ were comparable and as such, data are presented as absolute MEP amplitudes in the text and figures to facilitate comparison with other published research.

### Effect of PES on Corticomotor Excitability

**Figure 2:**
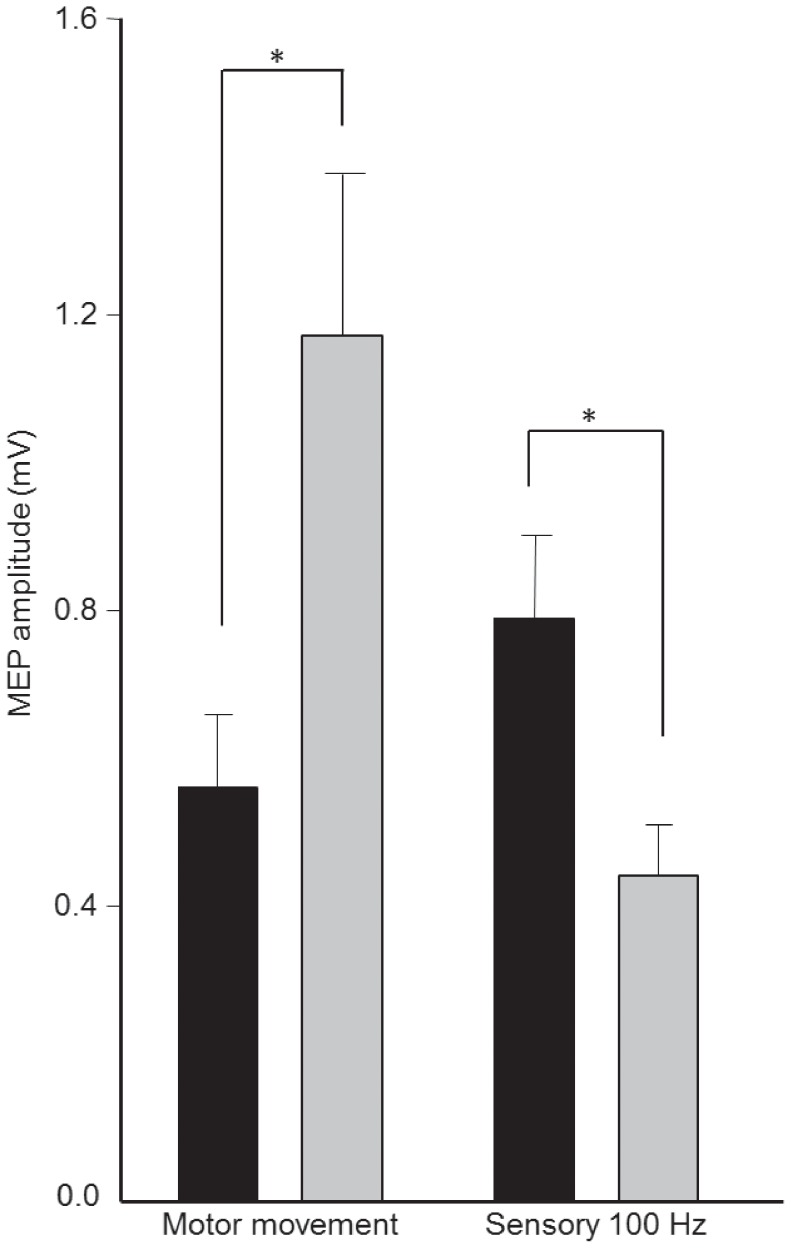
Group data (mean ± standard error) of amplitudes motor evoked potentials (MEP) before (black bars) and after (grey bars) “Motor Movement” and “Sensory 100 Hz” peripheral electrical stimulation (PES) to right abductor pollicis brevis muscle (APB). MEP amplitude increased following Motor Movement PES and reduced following Sensory 100 Hz PES. * p<0.05.

Motor and sensory PES paradigms induced different effects on corticomotor excitability (Interaction time × condition p<0.001). Motor PES applied to right APB increased MEP amplitudes (post-hoc pre vs. post p<0.001), whereas sensory PES suppressed MEP amplitudes (post-hoc pre vs. post p = 0.019; Figure 2). There was no difference in MEP amplitude between the two interventions at baseline (post-hoc sensory PES vs. motor PES pre intervention p = 0.24). However, the two interventions induced effects on corticomotor excitability that differed from each other following the 30-min stimulation period (post-hoc sensory PES vs. motor PES post intervention p<0.001).

### Effect of PES on Sensory Cortex Excitability

**Figure 3:**
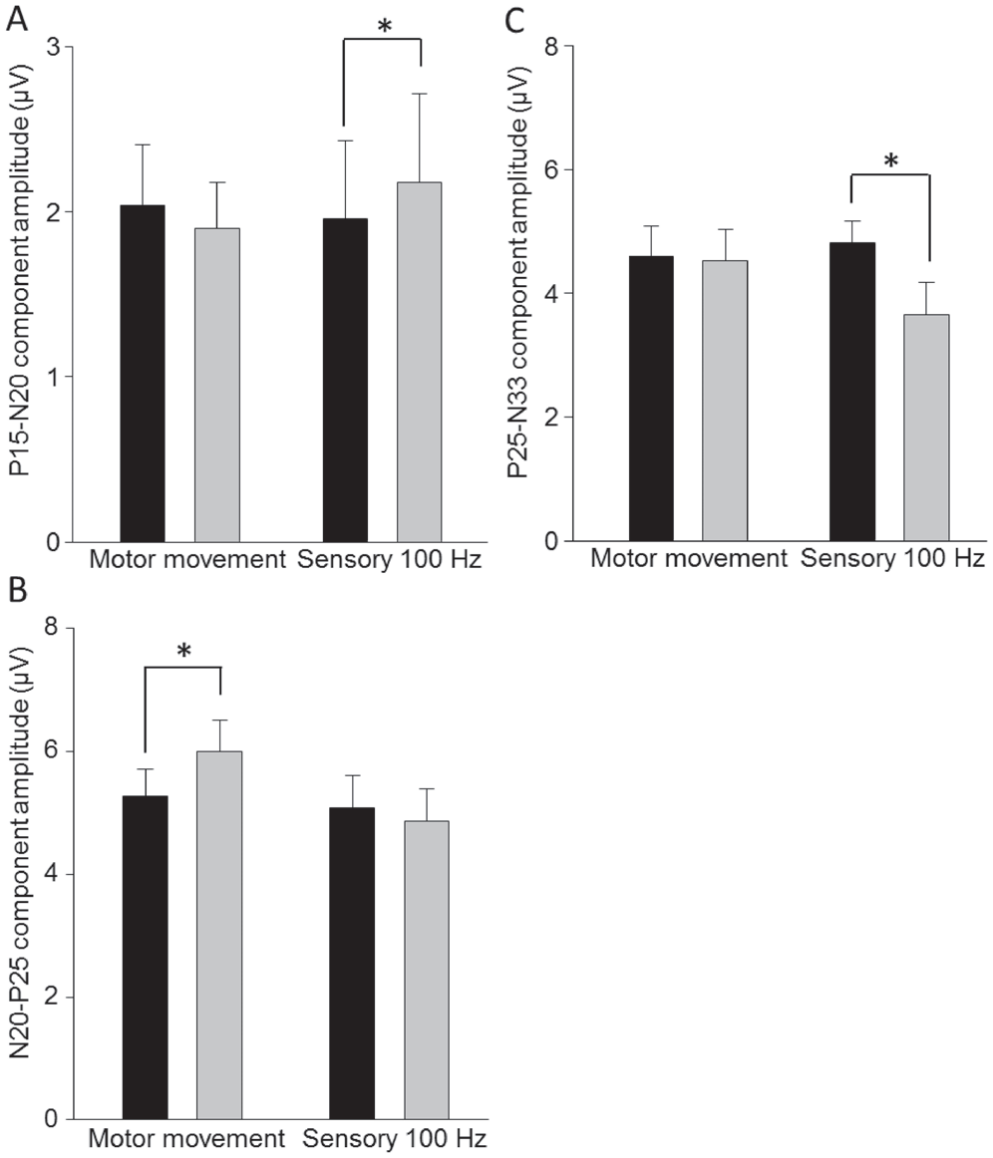
Group data (mean ± standard error) before (black bars) and after (grey bars) Motor Movement and Sensory 100 Hz peripheral electrical stimulation (PES) to the right abductor pollicis brevis muscle (APB) for the SEP components (a) P_14_-N_20_, (b) N_20_-P_25_ and (c) P_25_-N_33_. Motor Movement PES increased the amplitude of the N_20_-P_25_ component. Sensory 100 Hz PES increased the amplitude of the sub-cortical P_14_-N_20,_ and reduced the size of the P_25_-N_33_ component. * p<0.05.

There was no effect of either intervention on the spinal (N_13;_ main effect of time p = 0.32; Interaction time × condition p = 0.66) or peripheral (N_9_; main effect of time p = 0.40; Interaction time × condition p = 0.67) volley. Neither motor nor sensory PES induced a change in the latency of the N_20_ (main effect of time p = 0.74; Interaction time × condition p = 0.68), N_13_ (main effect of time p = 0.78; Interaction time × condition p = 0.48) or N_9_ (main effect of time p = 0.51; Interaction time × condition p = 0.53) components. Differential effects of *motor* and *sensory* PES on SEPs were observed for the P_14_-N_20_ (Interaction time × condition p = 0.039), N_20_-P_25_ (Interaction time × condition p = 0.032) and P_25_-N_33_ (Interaction time × condition p = 0.023) components. Following *motor* PES the N_20_-P_25_ increased (post-hoc pre vs. post p = 0.007, Figure 3b) but there was no change in the P_14_-N_20_ (post-hoc pre vs. post p = 0.34) or P_25_-N_33_ (post-hoc pre vs. post p = 0.77) components. Conversely, *sensory* PES increased the amplitude of P_14_-N_20_ (post-hoc pre vs. post p = 0.01, Figure 3a) and reduced P_25_-N_33_ (post-hoc pre vs. post p<0.001, Figure 3c). The N_20_-P_25_ component was unchanged by sensory PES (post-hoc pre vs. post p = 0.34).

**Figure 4:**
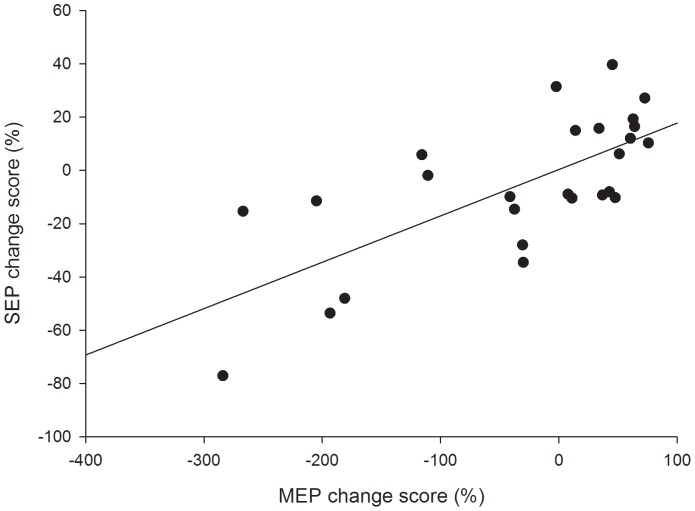
Linear regression between cortical SEP components (N_20_-P_25_ and P_25_-N_33_) and corticomotor excitability (MEP amplitude). Note the significant positive correlation (r = 0.71, p<0.001) between these parameters.

The magnitude and direction (increase or decrease) of the change in corticomotor excitability induced by sensory and motor PES was positively correlated with the change in the cortical SEP components (r = 0.71, p<0.001, Figure 4).

## Discussion

This study is the first to concurrently examine the influence of two PES paradigms on S1 and M1 excitability. Our data demonstrate increased excitability of the corticomotor pathway and increased amplitude of S1 responses, specifically of the early N_20_-P_25_ component, with PES at intensities sufficient to induce the movement of thumb abduction. Decreased excitability of the corticomotor pathway with PES applied at sub motor threshold (sensory) intensities was mirrored by a decrease in the N_25_-P_33_ component and an increase in subcortical processing, as evidenced by an increase in the P_14_-N_20_ component. These novel findings indicate that the excitability of S1 and M1 are co-modulated following PES and the direction of effect appears dependent on the combination of stimulus intensity and frequency.

PES at motor intensities is used to facilitate movement and improve function in a variety of pathologies including stroke and spinal cord injury [13]–[17]. Conversely, PES at sensory intensities (without muscle contraction), commonly termed “transcutaneous electrical nerve stimulation” (TENS), is used for pain relief and is effective for management of pain associated with rheumatoid arthritis, surgery and labour [18], [19]. We recently demonstrated increased corticomotor excitability when PES is applied at motor intensities but decreased when PES is applied at sub-motor threshold sensory intensities [4]; effects confirmed in the current study. The observed changes in corticomotor excitability likely occur at the motor cortex as both peripheral M-waves, indicative of excitability changes occurring at the neuromuscular junction and muscle, and measures of spinal/motoneurone excitability (H-reflex and F-waves) are unchanged following motor [20] and sensory PES [21]–[23]. Changes in motor cortex excitability following PES have been attributed to altered synaptic efficacy and associated long-term potentiation (LTP) or depression (LTD)-like mechanisms [24]. However, no study has attempted to examine how afferent input in the form of PES (in the absence of contraction) may drive reorganization in M1.

Afferent input plays a vital role in motor learning and its manipulation induces organisational changes in M1 [25]. For instance, removal of sensory input can change the cortical motor representation in a manner that is reversed when sensation is restored [26]. The presence of structural and functional connections between S1 and M1 suggests modulation of S1 excitability might result in similar changes in M1 excitability following PES. In support of this, in the current study changes in M1 excitability mirrored the changes in SEP components that relate to S1 function; motor PES increased, and sensory PES decreased both S1 and M1 excitability. Further, the magnitude and direction of the PES induced effects on corticomotor excitability were positively correlated with changes in S1 excitability. One explanation for our findings is that afferent information from PES is relayed to S1 via thalamo-cortical projections, activating or inducing a change in sensory processing and this provides the signal for LTP or LTD-like changes in M1. Cortico-cortical projections between S1 and M1 have been identified in animals and humans [27], [28] and these projections are topographically specific. Evidence from animal studies demonstrates that stimulation of S1 can induce LTP of motor cortical synapses probably through altered discharge of intracortical interneurons [5]. This mechanism may underpin the co-modulation of S1 and M1 observed here. To further clarify this mechanism, future studies should seek to examine intracortical inhibitory and facilitatory networks in response to PES at various stimulus intensities. However, direct connections also exist between the thalamic nucleus and M1 [29]–[31]. Thus, we cannot dismiss the possibility that afferent input from PES may relay directly to S1 and M1 via the thalamus, providing a stimulus for LTP or LTD-like changes in synaptic efficacy in both regions within a similar timeframe.

There is good evidence that the N_9_ component of the SEP represents conduction of the potential along the peripheral nerve, N_13_ in the cervical dorsal horn and P_14_-N_20_ in the cervicomedullary junction near the cuneate nucleus [7]–[9], [32]–[34]. The N_20_-P_25_ component represents arrival of the afferent volley in S1 and the P_25_-N_33_ is thought to represent processing of the afferent volley in S1 [7]–[9], [32]–[34]. Traditionally, the spinal cord has been considered an important site affected by sensory PES [35]. Yet, spinal N_13_ was unchanged by sensory PES in the current study. Consistent with previous studies, this suggests sensory PES does not inhibit electrically evoked spinal N_13_ activity [9], [34]. Further, consistent N_9_ and N_13_ amplitudes, regardless of stimulation type, indicate that altered SEP excitability in response to PES occurred at supraspinal levels, and these could be either sub-cortical or cortical.

Single electrical stimuli of increasing intensity have been shown to amplify afferent signals in the central nervous system (CNS) [32], [34]. This amplification occurs primarily at the level of the cuneate nucleus (measured as an increase in P_14_-N_20_) and is maintained at the level of S1. Application of sensory PES in the current study produced an increase in the size of the P_14_-N_20_ component, suggesting sensory PES as applied here did not alter expected amplification at the cuneate nucleus. However, consistent with previous reports [34], our findings indicate that amplification is suppressed at S1 (N_20_-P_25_ and P_25_-N_33_). The magnitude of the N_20_-P_25_ and P_25_-N_33_ SEP components reflect the size of the arriving synaptic input and responsiveness of the post-synaptic cell respectively [36]. As the size of the input arriving at S1 remained stable with sensory PES, suppression of S1 excitability is most likely explained by activation of post-synaptic inhibitory mechanisms [34]. This inhibitory response may drive reduced corticospinal output via S1-M1 cortico-cortical circuitry in response to sensory PES.

Several possibilities may explain the differential effect of sensory and motor PES on S1 and M1. First, corticomotor excitability is increased when motor PES is applied to a mixed nerve or over the muscle motor point, but identical PES protocols administered to digital nerves (consisting primarily of cutaneous afferents) fail to alter M1 excitability [37], [38]. These findings, in conjunction with those of the present study, suggest input from large-diameter afferents from *muscle* may be an important factor driving enhanced S1 excitability and subsequent LTP-like changes in M1 with motor PES. Second, a key feature of sensory PES is the bombardment of S1 with consistent afferent stimuli that presumably provide little or no useful information regarding sensory or motor function. It is possible that repeated, functionally irrelevant activation of S1 ‘gates’ or suppresses S1 excitability during sensory PES [36]. On the other hand, motor PES generates afferent input both from electrical stimulation of the afferent neurons and the “natural” input from the evoked movement, providing potentially “useful” information relating to movement. The N_20_-P_25_ and P_25_-N_33_ SEP components are thought to reflect processing related to kinaesthesia and position sense [39], [40]. Therefore, their enhancement (and the associated increase in corticomotor excitability) following motor PES may be important for modulating motor output.

### Conclusion

Excitability of primary sensory and motor cortical areas is co-modulated in response to PES, regardless of stimulus intensity and frequency. PES applied in a manner that induced strong thumb abduction increased S1 and M1 excitability, whereas PES at sensory intensities (below motor threshold) reduced S1 and M1 activity. These findings appear consistent with the hypothesis that reorganisation of M1 in response to PES is influenced by cortico-cortical projections between S1 and M1, a circuit that has been previously implicated in motor learning.
